# An innovative training based on robotics for older people with subacute stroke: study protocol for a randomized controlled trial

**DOI:** 10.1186/s13063-021-05357-8

**Published:** 2021-06-14

**Authors:** Elvira Maranesi, Roberta Bevilacqua, Mirko Di Rosa, Giuseppe Pelliccioni, Valentina Di Donna, Riccardo Luzi, Micaela Morettini, Agnese Sbrollini, Elisa Casoni, Nadia Rinaldi, Renato Baldoni, Fabrizia Lattanzio, Laura Burattini, Giovanni R. Riccardi

**Affiliations:** 1 Clinical Unit of Physical Rehabilitation, IRCCS INRCA, Ancona, Italy; 2 Scientific Direction, IRCCS INRCA, Ancona, Italy; 3 Unit of Geriatric Pharmacoepidemiology, IRCCS INRCA, Ancona, Italy; 4 Neurology Unit, IRCCS INRCA, Ancona, Italy; 5 Clinical Unit of Physical Rehabilitation, IRCCS INRCA, Fermo, Italy; 6 Medical Direction, IRCCS INRCA, Ancona, Italy; 7 Cardiovascular Bioengineering Lab, Department of Information Engineering, Università Politecnica delle Marche, Ancona, Italy

**Keywords:** Stroke patients, Gait training, End-effector gait rehabilitation

## Abstract

**Background:**

Stroke is a leading cause of disability, injury, and death in elderly people and represents a major public health problem with substantial medical and economic consequences. The incidence of stroke rapidly increases with age, doubling for each decade after age 55 years. Gait impairment is one of the most important problems after stroke, and improving walking function is often a key component of any rehabilitation program. To achieve this goal, a robotic gait trainer seems to be promising. In fact, some studies underline the efficacy of robotic gait training based on end-effector technology, for different diseases, in particular in stroke patients. In this randomized controlled trial, we verify the efficacy of the robotic treatment in terms of improving the gait and reducing the risk of falling and its long-term effects.

**Methods:**

In this single-blind randomized controlled trial, we will include 152 elderly subacute stroke patients divided in two groups to receive a traditional rehabilitation program or a robotic rehabilitation using G-EO system, an end-effector device for the gait rehabilitation, in addition to the traditional therapy. Twenty treatment sessions will be conducted, divided into 3 training sessions per week, for 7 weeks. The control group will perform traditional therapy sessions lasting 50 min. The technological intervention group, using the G-EO system, will carry out 30 min of traditional therapy and 20 min of treatment with a robotic system. The primary outcome of the study is the evaluation of the falling risk. Secondary outcomes are the assessment of the gait improvements and the fear of falling. Further evaluations, such as length and asymmetry of the step, walking and functional status, and acceptance of the technology, will be carried.

**Discussion:**

The final goal of the present study is to propose a new approach and an innovative therapeutic plan in the post-stroke rehabilitation, focused on the use of a robotic device, in order to obtain the beneficial effects of this treatment.

**Trial registration:**

ClinicalTrials.gov NCT04087083. Registered on September 12, 2019

## Background

According to data from “World Population Prospects: the 2017 Revision,” the number of older persons is expected to more than double by 2050 and to more than triple by 2100, rising from 962 million globally in 2017 to 2.1 billion in 2050 and 3.1 billion in 2100 [1]. Globally, the number of persons aged 80 or over is projected to triple by 2050 [2].

Stroke is a leading cause of disability, injury, and death in elderly people and represents a major public health problem with substantial medical and economic consequences [3]. The incidence of stroke rapidly increases with age, doubling for each decade after aged 55. Among adults aged 35 to 44 years old, the incidence of stroke is 30 to 120 of 100,000 per year, and for those ages 65 to 74, the incidence is 670 to 970 of 100,000 per year [4]. Annually, 15 million people worldwide suffer from stroke. Of these, 5 million die and another 5 million are left permanently disabled [5]. Three months after stroke, 20% of individuals remains wheelchair bound and 70% walks at reduced velocity [6]. Gait impairment is one of the most important problems after stroke and it is associated with difficulties in performing daily activities and in maintaining indoor and outdoor mobility. For this reason, improving walking function is often a key component of any rehabilitation program. To achieve this goal, a specific task-repetitive training seems the most promising one since, by increasing therapy dosage, intensity, and number of repetitions, the plasticity and the functional recovery are promoted [7–9]. To achieve this goal, gait machines were developed following exoskeleton [10, 11] or end-effector principles [12]. In the exoskeleton systems, there is a one-to-one correspondence between robots and human joints, and each single joint is guided along a preprogrammed trajectory. The end-effector systems use footplates or handles to generate a motion of the limb in space, as movements are generated from the most distal segment of the extremity and no alignment between patient-robot joints is required. On the basis of the support provided, the electromechanical devices can be also divided into inactive, passive, active-assisted, resistive, and interactive devices [13].

Several trials have been published regarding the use of these devices in stroke patients, but no differences were found between the two types of robotic gait machines [14]. Despite this evidence, some studies [15–18] underline the efficacy of robotic gait training based on end-effector technology, for different diseases. In particular, stroke patients, exposed to only robot-assisted end-effector-based gait training, showed significant improvements in global motor performance, gait endurance, balance and coordination, lower limb strength, and even spasticity.

Due to the incidence of the gait impairments after stroke and the low degree of acceptance of exoskeletons [19], we strongly believe that the efficacy of robotic rehabilitation treatments based on end-effector technology has to be deeply investigated.

### Study aims and objective

This study aims to evaluate an innovative rehabilitation treatment based on robotics, for the older subacute stroke patients, designed to improve the gait and to reduce the risk of falling. The treatment involves the use of the G-EO system (Reha Technology, Switzerland), an end-effector technology that simulates floor walking and stair climbing (up and down). The study objectives are:

1. To evaluate the effect of the rehabilitation treatment on the decrease of falling risk, evaluating balance and gait performance, of the elderly subacute stroke patients, as a result of the use of the G-EO system, at the end of the treatment and at 6 months, 1 year, and 2 years of follow-up
2. To evaluate the effect of the gait improvement by the gait speed evaluation on rehabilitation treatment on the gait speed of the elderly subacute stroke patients, as a result of the use of the G-EO system, at the end of the treatment and at 6 months and 1 and 2 years of follow-up
3. To evaluate the effect of the rehabilitation treatment on the fear of falling of the elderly subacute stroke patients, as a result of the use of the G-EO system, at the end of the treatment and at 6 months and 1 and 2 years of follow-up

In addition, the study design will include using standardized questionnaires and instrumental gait analysis, in order to collect data on the improvements with a mix-method approach.

## Methods/design

### Trial design

This study is a single-blind (outcome assessors) randomized controlled trial. In particular, the single-blind research method consists in not letting the researcher know the type of rehabilitation path assigned to each participant. Each subject will be assigned an identification code that will be known only to the assessor who will carry out the recruitment, the end of treatment, and the follow-up assessments. A total of 152 elderly subacute stroke patients will be recruited and randomly divided into two groups, to receive a traditional rehabilitation program or a robotic rehabilitation using the G-EO system in addition to the traditional therapy. Assessments will be performed at baseline, at the end of treatment, and 6 months, 1 year, and 2 years from the end of the treatment.

### Study setting

The study will be conducted at the Clinical Unit of Physical Rehabilitation, IRCCS INRCA, in the Ancona and Fermo branches, Italy. Assessments and treatments will be conducted in the robotics laboratories.

### Trial status

The protocol version number is 1 in date July 15, 2019.

At the time of the submission of this study protocol, data collection is ongoing; the first patient was enrolled on July 13, 2020. We expect to finish recruiting in 2 years.

### Participants

The inclusion criteria are:

- Aged 65 and over
- Capacity to consent
- Ischemic or hemorrhagic stroke within 3 months to the recruitment, proven by computerized axial tomography or nuclear magnetic resonance
- Functional Ambulation Category (FAC) score ≤ 2
- Rankin Scale score ≤ 3
- Complete communication and comprehension skills, assessed during the objective examination
- Ability to stand upright, supported or unsupported, for 1 min [20]

The exclusion criteria are:

- Failure to meet the inclusion criteria
- Concomitant participation in other studies
- Severe hypertonus of the hip, knee, and ankle of the paretic leg with a modified Ashworth scale score ≥ 3
- Severe hip, knee, or ankle contracture or orthopedic problem affecting ambulation that would preclude passive range of motion of the paretic leg
- Deep vein thrombosis of the lower limbs
- Other cognitive, motor, and sensory deficits that negatively condition robotic training
- Treatment of spasticity of the lower limb within 3 months to the start of the study or during the study
- Lack of written informed consent
- Clinical Dementia Rating (CDR) score ≥ 3
- Severe systemic diseases with life expectancy < 1 year
- Patients unable to follow-up

### Sample size

In the study of Tramontano et al. [21], the Performance-Oriented Mobility Assessment (POMA) was evaluated in two groups of stroke patients, the cases were treated through an intervention based on visual-spatial training, and the assessments were detected in 2 stages (baseline and follow-up).

POMA [22], a test widely used to assess walking ability and associated with equilibrium, was used to calculate the sample size [23]. Assuming a small effect size of 10% [24], a statistical power of 80%, a significance level of 0.05, a correlation among repeated measures of 0.5, and that sphericity is exactly met, it is estimated that the overall sample size needed to capture this effect size is of 122 subjects, two groups, and 5 repeated assessments (a baseline and 4 follow-ups) in an ANOVA model within-between interactions. Even assuming a 20% drop-out rate, the total number required would be 152 subjects (76 for each arm). The GPower (Heinrich-Heine-Universität Düsseldorf, Düsseldorf, Germany) software was used to estimate this sample size.

For the secondary outcomes, we assumed an improvement of 12% and 15% for gait speed and fear of falling, respectively. These increases have been hypothesized on the basis of articles [25, 26] that identify an improvement in these parameters using a treatment comparable to that used in this protocol.

### Recruitment

Patients will be selected by the outpatient department at the Clinical Unit of Physical Rehabilitation, IRCCS INRCA, in the Ancona and Fermo branches. These patients will be contacted to schedule a visit with the physician. Once the compliance with the inclusion and exclusion criteria of the study has been verified and the informed consent has been obtained in triplicate, the doctor will proceed with the baseline evaluation and with acquisition of gait assessment parameters through gait analysis at the Movement Analysis Laboratory of the Clinical Unit of Physical Rehabilitation of the Ancona branch.

A randomization technique based on a single sequence of random assignments will be used. A list of random numbers generated by the computer will be used and subjects will be assigned a number based on their order of inclusion in the study. The sequence will be saved to a password-protected spreadsheet. According to this technique, the 152 subjects will be randomly assigned to one of the 2 study groups: the control group and the technological intervention group. After the patient is assigned to one of the two groups, he will be given a code by which the physiotherapist will understand whether the patient should receive traditional or experimental treatment. The randomization was organized by using opaque envelopes: participating physicians are given randomly generated treatment allocations within sealed opaque envelopes. Once a patient has consented to enter a trial, an envelope is opened and the patient is then offered the allocated treatment group. The randomization schedule and coding of group allocations will not be accessible to the researcher conducting the assessments. According to the intention to treat (ITT) principle, subjects who were enrolled but withdrawn from the study will not be replaced. Researchers will be blinded to prevent observation bias. Statistical analyses will be conducted by a statistician, who will be blind to group allocation prior to analysis.

At the end of the treatment and after 6 months, 1 year, and 2 years, the patients will be contacted again to schedule subsequent follow-up visits and upgrades.

Recruitment will run from August 2019 to August 2021.

### Intervention

For the study, post-hospitalization subjects will be taken into consideration, after 4 weeks from the hospitalization in the Clinical Unit of Physical Rehabilitation, IRCCS INRCA, in the Ancona and Fermo branches, who have already received the standard treatment. Twenty treatment sessions will be conducted, divided into 3 training sessions per week, for 7 weeks. The control group will perform traditional therapy sessions lasting 50 min. The technological intervention group will carry out 30 min of traditional therapy and 20 min of treatment with a robotic system. Cardiac activity monitoring is planned during robotic treatments.

Individual participants must complete at least 80% of the sessions. Recovery of 3 sessions will be possible.

All patients included in the study will perform traditional rehabilitation treatments, consisted in:

- Appropriate positioning in bed and chair
- Neuromotor treatment for the trunk control recovery and movement in bed
- Exercises for verticalization, load balancing, and balance control from an upright position
- Walking training with correct use of aids
- Neuromotor treatment for the recovery of functional motility of the upper limb
- Improved autonomy in daily life activities with the use of aids, if necessary

The robotic treatment consists of using the G-EO system, an advanced robot-assisted, end-effector device in gait rehabilitation. The G-EO system allows to simulate both floor walking and stair climbing. The G-EO floor walking movement is generated by the motion of two footplates and allows to select different execution modality: active mode, active-assistive mode, and passive mode. The standard procedure consists of 5 sessions performed using the passive mode and 15 using the active-assistive mode. Taking into account that a personalized approach will be carried out, the therapy may be modified in based of patients’ needs. Cardiac activity monitoring is planned during robotic treatments.

Adverse events, although unlikely, could be related to the use of technology such as falls and/or pain at knees or ankles. In this case, these effects are further minimized by the presence of the lashing.

### Outcomes

All outcome measures follow a standardized operating procedure. Table 1 shows the primary outcome and the secondary outcomes with the expected result at the end of the treatment. The expected improvement was derived from the analysis of similar studies [24], collected for the evaluation of the sample size for each outcome.

**Table 1:** Outcomes and clinical assessments

Outcome(s)	Clinical assessment	Expected improvement at the end of treatment (%)
Primary: Decrease of falling risk	POMA	10
Secondary: Gait improvement (increase in walking speed)	Gait speed (gait analysis)	12
Secondary: Decrease of fear of falling	FES-I Short form	15

*POMA* Tinetti’s Scale or Performance-Oriented Mobility Assessment, *FES-I* Short Falls Efficacy Scale—International

Further evaluations will be carried out as follows:

- Length and asymmetry of the semi-step, through instrumental gait analysis
- Walking and functional status through the Functional Ambulation Category and Barthel Index scale
- Acceptance of the technology, through a UTAUT questionnaire

A summary of all data collected and when these are collected is provided in Table 2.

**Table 2:** Schedule of assessment and outcome measures

Outcome	Clinical assessment	R	T1	FW1	FW2	FW3
Cognitive state	Mini-Mental State Examination	✓				
Gait parameters	Functional Ambulation Category	✓	✓	✓	✓	✓
Disability state	Rankin Scale	✓				
Cognitive state	CDR	✓				
Functional state	Barthel Index	✓	✓	✓	✓	✓
Spasticity state	Modified Ashworth scale	✓				
Quality of life	SF-12 Health Survey	✓	✓	✓	✓	✓
Sociodemographic characteristics	Check-list	✓				
Motor ability	Motricity index	✓	✓	✓	✓	✓
Attitude to technology	Assistive Device Predisposition Assessment – Scala E	✓				
Fall risk	Scala di Tinetti	✓	✓	✓	✓	✓
Gait parameters	Gait analysis + instrumental postural analysis	✓	✓	✓	✓	✓
Fear of falling	Short Falls Efficacy Scale—International	✓	✓	✓	✓	✓
Acceptance of technology	Psychosocial Impact of Assisted Device Scale (PIADS)		✓			

*R* recruitment, *T1* end of treatment, *FW1* first follow-up at 6 months since the end of treatment, *FW2* second follow-up at 1 year since the end of treatment, *FW3* third follow-up at 2 years since the end of treatment

#### Mini-Mental State Examination (MMSE)

The MMSE was designed as a clinical method for grading cognitive impairment. The score ranges from 0 to 30: scores ≥24 indicate normality, between 18 and 23 indicate mild cognitive impairment, between 11 and 17 average cognitive deficits, and scores ≤10 severe cognitive impairments. The reported score is corrected for age and education [27].

#### Rankin Scale (RA)

It is a simple scale for the evaluation of the outcomes following the stroke. Reliability is well defined. The individual categories are essentially based on patient mobility. There are 6 grades of classification from 0 to 5, where 0 means independence [28].

#### Barthel Index (BI)

BI is an ordinal scale used to measure a subject’s performance in everyday life activities. The index analyzes ten variables that describe the activities of daily life and mobility. Each item is assigned a score between 0 and 10 depending on the degree of patient’s functionality: full, reduced, or no functionality. A high overall score is associated with a greater probability of being able to live at home independently after discharge from the hospital [29].

#### Functional Ambulation Categories (FAC)

The scale is used to classify the severity level of gait disturbances in neurological disorders. It provides a hierarchical classification from level 0 (impossible walking) to level 5 (no limitation) [30].

#### Modified Ashworth scale (SA)

The scale carries out the assessment of spasticity/hypertonus. The scores range from 0 to 4, 0 no spasticity and 4 rigid limbs in flexion or extension [31].

#### SF-12 Health Survey (SF-12)

The SF-12 questionnaire was originally developed in the USA to provide a short alternative form to the SF-36 questionnaire. The SF-12 is composed of 12 items that produce two measurements related to two different aspects of health: physical health and mental health. The subject is asked to answer on how he feels and how he is able to carry out the usual activities, evaluating the current day and the 4 previous weeks [32].

#### Tinetti’s Scale or Performance-Oriented Mobility Assessment (POMA)

Tinetti scale is a tool used to evaluate balance and gait performance. The test is used clinically to determine the mobility status of a subject or to assess changes in balance and gait time. The total POMA (POMA-T) consists of two sub-scales: the balance evaluation scale (“balance scale” or POMA-B) and the gait evaluation scale (“gait scale” or POMA-G). The maximum score is 28 points: in detail, the maximum score of the POMA-B is 16, while for the POMA-G the maximum score is 12 [22].

#### Motricity index (MI)

The scale shows a significant correlation between lower limb scores and dynamometric measurements. The total score ranges from 0 to 100. It provides 3 items with a score between 0 and 33: 0 no movement, 33 movement performed with normal force [33].

#### Short Falls Efficacy Scale—International (FES-I-Short)

The scale measures the “fear of falling.” The scale can be self-administered or administered during the interview. The cutoffs for the fear of falling are divided as follows: a score between 7 and 8 indicates a low concern, between 9 and 12 a moderate concern, and between 14 and 28 a high concern [34].

#### Psychosocial Impact of Assistive Devices Scale (PIADS)

It is a self-completed questionnaire by the user and it assesses the impact that the device has on the person. Through 26 questions, it tries to detect how the device has brought about a perception of change with respect to one’s availability for new experiences (6 questions), skills (ability to cope with daily activities and challenges—12 questions), and self-esteem (security and self-confidence—8 questions). Every question is answered on a visual scale marked by −3 (the device has strongly limited my independence) to + 3 (the device has greatly improved my independence) [35].

#### Assistive Device Predisposition Assessment (ATDPA)

The purpose of the tool is to assess user expectations about technological devices [36].

#### Clinical Dementia Rating Scale (CDR)

This questionnaire assesses the patient’s dementia status. The CDR is a 5-point scale used to characterize six domains: memory, orientation, judgment and problem solving, business, home and hobby, and personal care [37].

#### Gait analysis and instrumental postural analysis

Gait analysis is the systematic study of human locomotion, augmented by instrumentation for measuring body movements, body mechanics, and the activity of the muscles [38]. Gait analysis is performed on the selected patients at the Gait Analysis Laboratory in the Department of Physical Rehabilitation at branch of IRCCS INRCA Ancona. Instrumented gait analysis is performed using BTS GAITLAB (BTS Bioengineering, Italy) system with six infrared cameras (100 Hz) and 2 force plates (50 Hz). The system is used to acquire both kinematic and kinetic data. Three-dimensional kinematic data are recorded with the help of 22 reflective infrared markers using Helen Hayes protocol [39]. The floor-mounted force plates are used to acquire the kinetic data. The subjects walked at a self-selected speed. The instrumental postural analysis studies the complex control system that must keep the center of gravity constantly in a balanced position. This analysis is carried out through an advanced system composed of a camera for video recording and a platform with 2 triaxial sensory plates (Podium, BTS Bioengineering, Italy). With this system, it is possible to simultaneously visualize the three components of force: vertical, antero-posterior, and lateral. It allows to evaluate, in augmented reality, the symmetry of the load and the trend of the pressure center.

### Data management

Personal data collected during the trial will be handled and stored in accordance with the General Data Protection Regulation (GDPR) 2018. Use of the study data will be controlled by the principal investigator. All data and documentation related to the trial will be stored in accordance with applicable regulatory requirements and access to data will be restricted to authorized trial personnel.

### Data analysis

The ITT approach will be adopted. Descriptive statistics will be used to describe the patients’ characteristics at baseline, compare the POMA values, the FES-I scores, and the gait speed in the two groups. Continuous variables will be expressed as mean values ± standard deviation. Categorical variables will be expressed as absolute values and relative frequencies. Independent samples’ t-tests and chi-squared test analyses will be used to compare both baseline characteristics and outcomes between groups. The primary efficacy analysis will be based on within-between group differences in the primary outcome measures (decrease in fall risk measured by POMA score) following 6 months of training with the G-EO system. The secondary outcome progression will be evaluated by comparing the measures of gait speed and FES-I scale from baseline to 6-month follow-up.

Comparisons for both primary and secondary outcomes will be repeated at each time point. Finally, repeated measures mixed models with interactions for each outcome will be performed in order to evaluate the effectiveness of the intervention. All tests for significance used a two-sided p-value of 0.05. Data were analyzed with STATA 15.1 (StataCorp, College Station, TX, USA).

## Discussion

The aim of this protocol is to evaluate an innovative rehabilitation treatment of the elderly subacute stroke patients, designed to improve the gait and to reduce the risk of falling through the use of the G-EO system, an end-effector technology that simulates floor walking and stair climbing. Moreover, the study aims to evaluate the effect of the rehabilitation treatment on the balance and gait performance, on the gait speed, on the fear of falling, and on the quality of life, acceptance of technology, and improvement of functional status as results of the use of the G-EO system.

The target population of the study consists of stroke patients within 3 months from the event, who need physical assistance from another person to walk in the form of continuous or intermittent manual contact, or who cannot walk independently. A population with these characteristics was chosen because the scientific evidence shows that patients with highly impaired walking ability are able to have greater benefits from robotic rehabilitation than those patients who are already walking independently [40]. A total of 152 elderly subacute stroke patients will be recruited and randomly divided into two groups, to receive a traditional rehabilitation program or a robotic rehabilitation using the G-EO system in addition to the traditional therapy.

We will compare the results of the several outcomes (falling risk, gait improvement in terms of walking speed, length and asymmetry of the step, fear of falling, walking and functional status, and acceptance of the technology) between the two groups in order to verify the efficacy of the robotic treatment. The study considers three different follow-ups: at 6 months, 1 year, and 2 years from the end of the treatment to monitor the conservation of any improvements obtained.

In fact, the final goal of the present study is to propose a new approach and an innovative therapeutic plan in the post-stroke rehabilitation, focused on the use of robotic device, in order to obtain the beneficial effects of this treatment.

The dissemination program will involve peer-reviewed journal and national and international conferences. Moreover, the results will be disseminated to all participants.

## Data Availability

The datasets generated, used, and analyzed during the trial and its preceding pilot trial are or will be available from the corresponding author upon reasonable request.

## Abbreviations

ATDPA: Assistive Technology Device Predisposition Assessment
BI: Barthel Index
CDR: Clinical Dementia Rating Scale
FAC: Functional Ambulation Categories
FES-I-Short: Short Falls Efficacy Scale—International
GDPR: General Data Protection Regulation
ITT: Intention to treat
MI: Motricity index
MMSE: Mini-Mental State Examination
PIADS: Psychosocial Impact of Assistive Devices Scale
POMA: Tinetti’s Scale or Performance-Oriented Mobility Assessment
RA: Rankin Scale
SA: Modified Ashworth scale
SF-12: SF-12 Health Survey
